# Impact of HCV cure on systemic inflammation and bone density, quality, and turnover

**DOI:** 10.3389/fimmu.2025.1626875

**Published:** 2025-11-28

**Authors:** Christina K. Psomas, Elsa Lellouche-Slama, Susan Langan, Mark Sulkowski, Kendall F. Moseley, Jenny Pena-Dias, Smita Khati, Jing Sun, Alison G. Abraham, Todd T. Brown

**Affiliations:** 1 European Hospital Marseille, Marseille, France; 2 Sorbonne University, Paris, France; 3 Johns Hopkins University, Baltimore, MD, United States; 4 University of Colorado, Aurora, CO, United States

**Keywords:** HCV cure, HCV-HIV coinfection, DAA, inflammation, bone outcomes, DXA, P1NP, CTX

## Abstract

**Background:**

Chronic hepatitis C virus (HCV) infection has been associated with osteoporosis and fragility fracture, which may be mediated by increased systemic inflammation, especially in HIV-HCV co-infection. Treatment with direct-acting antivirals (DAA) eradicates HCV and decreases inflammation, but the impact on bone parameters has not been studied.

**Methods:**

We recruited individuals with HCV infection with and without HIV co-infection who initiated DAA, and a demographically matched reference group without HIV or HCV, on whom dual-energy X-ray absorptiometry (DXA) scans were performed at baseline. All participants with HCV who displayed sustained virologic response were included and underwent a follow up visit with DXA between 52 and 134 weeks after baseline to measure bone mineral density at the lumbar spine (LS), femoral neck (FN), and total hip (TH), as well as the trabecular bone score (TBS), a bone quality measure. Bone turnover markers (BTM) and inflammatory biomarkers were also measured at baseline and the follow-up visit. We compared the change in inflammatory biomarkers and bone outcomes over time between the groups.

**Results:**

The group with HCV (41 mono-infected, 18 people with HIV/HCV co-infection) had a median age of 54 years; 61% were male and 76% were African American. Population characteristics were similar in the reference group (n=53). At baseline, soluble receptor for TNFα 1 and 2 (sTNFR-1/sTNFR-2) and sCD163 concentrations, but not interleukin-6, were higher in participants with HCV infection compared to the reference group. After a median follow-up period of 60 weeks, HCV cure was associated with decreases in sTNFR1, sTNFR2 and sCD163 concentrations. However, we observed no statistically significant changes in BMD, TBS or BTM after HCV treatment compared to the reference population. Among those with HCV, participants with HIV co-infection showed a significant increase in the bone formation marker P1NP (p<0.007) and trends toward greater increases in LS and TH BMD (p<0.08 for both) after HCV cure.

**Conclusion:**

HCV cure using DAA was associated with a decrease in systemic inflammation without changes in bone parameters. The significant increase in bone formation markers observed in HIV/HCV co-infected individuals suggests potential bone recovery in this high-risk group, warranting investigation in larger long-term studies.

## Introduction

Approximately 2.2 million US adults have chronic hepatitis C virus (HCV) infection (1) and 50 million individuals have HCV worldwide, of whom 64% remain undiagnosed (2). Chronic hepatitis C infection is a well-established cause of liver-related morbidity ranging from hepatocellular insufficiency to cirrhosis in up to 10-15% of patients, with a risk of further progression to hepatic decompensation, liver failure or hepatocellular carcinoma of up to 30% of these patients (3, 4). Extra-hepatic manifestations (EHM) are also frequent and affect multiple organ systems. In a meta-analytic review, the pooled prevalence of EHM was high, with approximately 30% of those with HCV having mixed cryoglobulinemia, 10% having chronic renal disease, 15% having diabetes mellitus, 12% having Sjögren’s syndrome and 25% having depression (5–7). Treatment with direct-acting antivirals (DAA) has dramatically reduced EHM-related mortality (8, 9), and the prevalence of these conditions (10). Among endocrine and metabolic disorders, chronic HCV infection has been associated with reduced bone mineral density (BMD) (11) and osteoporosis (12, 13) and has been identified as a risk factor for fragility fracture (14). Low bone mineral density affects up to 40% of non-cirrhotic patients chronically infected with HCV (15). In persons with chronic HCV infection, excess fracture risk may be as high as 53% (13). Osteoporotic fractures account for increased morbidity and mortality, making their prevention a priority when treating patients with chronic HCV infection.

Moreover, evidence shows that patients with HIV/HCV co-infection have a higher prevalence of osteoporosis (16, 17) and higher fracture risk compared not only to individuals without HIV or HCV, but also to participants with HIV or HCV mono-infection (18–20). In this population with co-infection, estimated osteoporosis prevalence is 22% (21) and patients have a three-fold increased incidence of fractures compared with uninfected individuals.

Although the exact mechanisms by which the hepatitis C virus impacts bone health remain unclear, findings by Bedimo et al. suggest that alterations in the bone microarchitecture occuring during chronic infection might explain the higher incidence of osteoporotic fractures (22, 23). Those alterations could result from increased bone turnover in the context of systemic inflammation, as well as chronic liver disease and hepatic osteodystrophy, which may affect bone metabolism via pathways independent of systemic inflammation, such as vitamin D or IGF-1. In addition, common risk factors for osteoporosis frequent in this population such as low body weight, smoking, and alcohol/drug abuse, may increase the risk of fragility fractures (24). Among those with HIV/HCV co-infection, additional factors may be present that impact bone heath, including residual inflammation related to chronic HIV infection (even with a suppressed viral load) and to specific antiretroviral therapies, such as tenofovir disoproxil fumarate (TDF).

Introduced in 2015, DAA result in HCV cure in more than 95% of patients after 8–12 weeks of treatment, defined by sustained virologic response (SVR). HCV cure with DAAs has been shown to reduce liver fibrosis and inflammation (25, 26). If osteoporosis and increased fracture risk are due to systemic inflammation or hepatitis C virus’ direct impact on bone metabolism, one could hypothesize that HCV cure could improve BMD and other bone health parameters. It is also possible that the impact of HCV on bone is irreversible or may take time to reverse. Chronic exposure to a pro-inflammatory environment in the setting of HCV may cause permanent structural changes or persistent osteoblast/osteocyte dysfunction that may not recover immediately after inflammation resolves. Previous studies examining the effects of HCV treatment using interferon + ribavirin +/- DAA or sofosbuvir + ribavirin on bone parameters have had mixed results (27–30). Both interferon and ribavirin may have direct effects on bone. Interferons type I and II may influence bone turnover by preventing excessive osteoclastogenesis (31, 32) and ribavirin may increase osteoclast formation thereby increasing bone resorption (33).

The objective of this study was to investigate whether the changes in bone mineral density and bone quality parameters (eg, bone turnover and microarchitecture) after successful HCV treatment with DAA differed from a matched control population without HIV or HCV. We also sought to understand how DAA impacted inflammatory markers especially in people with HIV and HCV co-infection. We hypothesized that successful HCV eradication with DAAs would reduce systemic inflammation, leading to improvements in BMD, bone quality, and turnover markers, particularly in HIV/HCV co-infected individuals.

## Materials and methods

### Study sample

Between October 2016 and November 2020, adults with chronic HCV infection who intended to receive curative treatment with DAA were recruited into a longitudinal study from the local infectious disease clinics in Baltimore, Maryland, USA. During the same period, adults without HIV infection (negative antibody testing within 6 weeks) or HCV infection (negative HCV antibody or undetectable HCV viral load without HCV treatment) were recruited from local clinics, from friends and family of participants, and by word of mouth to serve as a reference population. This reference population was frequency matched to those with HCV infection by age, race, and sex. Persons with weight > 300 pounds (weight limit of densitometer), with a history of receiving osteoporosis treatment, with a history of prior DAA use, or with a history of end-stage liver or kidney disease were excluded. All persons provided informed consent, and the study was approved by the Johns Hopkins School of Medicine Institutional Review Board.

### Study procedures

Participants underwent a baseline evaluation in the morning which included medical history questionnaires, a fasting blood draw, and BMD testing with dual-energy X-ray absorptiometry (DXA). For those with HCV infection, this baseline visit was ideally scheduled within 6 weeks of starting DAA. At 52 weeks after the baseline visit, participants returned for a follow-up visit in which phlebotomy and the DXA scan were repeated. HCV viral load was measured at an interim visit at 12–24 weeks and at the final follow-up visit to determine whether the participant achieved a sustained virologic response (SVR or HCV cure). Because of constraints imposed by the COVID-19 pandemic, the allowable follow-up time was extended to 30 months, and the interval between data collection was considered in all statistical analyses.

### Additional assessments

Medical history questionnaires included ascertainment of the prevalence of common comorbidities (eg diabetes, hypertension, cardiovascular disease), concomitant medications, and questions related to bone health, including use of vitamin D supplementation, estimated calcium intake, corticosteroid use > 3 months, and a parental history of hip fracture. Liver disease severity was assessed at baseline by transient elastography with use of FibroScan machine (Echosens, ON, Canada) that measures the velocity of a shear wave propagating through the liver (Kirk GD, Astemborski J, Mehta SH, 2009). Cigarette smoking was characterized as “current” vs “not current”. Hazardous alcohol use was assessed using the Alcohol Use Disorders Identification Test (AUDIT-C) (34, 35). and was defined as a score ≥ 4 for men and ≥ 3 for women. Physical activity was assessed using the International Physical Activity Questionnaire (36), and activity level was categorized as low, moderate, or high (37).

### Laboratory measurements

Serum samples were frozen and stored at -80 °C until analysis at the Advanced Chemistry Laboratory of the Johns Hopkins Institute of Clinical and Translational Research (Dr Neal Fedarko, Director). Serum interleukin-6 (IL-6) was measured using high-sensitivity Quantikine kits (R&D Systems) with a limit of detection (LOD) of 0.039 pg/mL and inter-assay coefficients of variation (CV) of 5.13% and intra-assay CV of 4.97%. Serum soluble receptor for TNFα 1 and 2 (sTNFR-1 and sTNFR-2) were measured using DuoSet ELISA kits (R&D Systems, Minneapolis, MN, USA) with a LOD of 1.2 pg/ml for sTNFR-1 and 2.3 pg/ml for sTNFR-2 and an inter-assay CV of 2.84% and 9.46%, respectively, and intra-assay CV of 2.56% and 2.55%, respectively. sCD163 was measured with ELISA kits (R&D Systems, Minneapolis, MN, USA) with a LOD of 613 pg/ml and inter-assay CV of 5.74% and intra-assay CV of 4.33%. Measurements were performed in duplicate and repeated if the measures differed by more than 15% or were out of the measurable range. The average of the two values in duplicate was used for analyses. Bone turnover markers (BTMs) included bone formation marker, amino-terminal propeptide of type 1 procollagen (PINP) and bone resorption marker, C-terminal telopeptide of type I collagen (CTX). CTX was measured using an enzyme-immunosorbent assay (Osteometer BioTech, Herlev, Denmark) and P1NP was measured using radio-immune assay (Immunodiagnostic Systems, Tyne & Wear, UK). Median intra-assay CVs were 8.15% and 4.14%, respectively, and the median inter-assay CVs were 0.01% and 8.09%, respectively. 25(OH)D was measured using radioimmunoassay (DiaSorin, Stillwater, Minnesota) and the CVs for these assays are 5.2% (intra-assay) and 7.9% (inter-assay). HCV RNA was measured with the Roche 6800 quantitative assay with a LOD of 15 IU/mL.

### Bone measurements

Body mass index (BMI) was calculated for all participants. bone mineral density (BMD) was assessed using DXA (Hologic Horizon, Marlborough, MA) at the lumbar spine and hip (total and femoral neck) by one of two certified examiners in the Johns Hopkins Clinical Research Unit. A single machine was used for all assessments which was calibrated daily. T-scores were calculated from the site-specific BMD measures using the White, young, female, database as a reference population for T-scores per International Society for Clinical Densitometry recommendations (38). DXA was also used to assess trabecular bone score, a measure of trabecular bone microarchitecture extracted from the DXA lumbar spine images (39) using TBS iNsight software (Medimaps, Geneva, Switzerland).

### Statistical analysis

We initially aimed to recruit 40 persons in each group, which would have had 90% power to detect a 1.7% difference in the change of hip BMD between persons with HCV initiating DAA and persons without HCV infection, approximately 2/3 of the effect size observed with interferon/ribavirin treatment in a clinical trial in HCV monoinfected persons (28). However, due to a higher-than-expected rate of loss to follow-up, exacerbated by COVID-19 pandemic challenges, we increased recruitment beyond these initial goals.

Independent t-tests were performed on normally distributed continuous variables to compare differences by HCV Status or HCV/HIV Status. Chi-squared or Fisher’s exact tests were used to compare categorical variables by HCV or HCV/HIV status. Inflammatory biomarkers and BTMs were normalized by log_2_ transformation. Other outcome measures that were not normally distributed were log_10_ transformed before analyses. Between-group comparison of baseline inflammatory markers and bone outcomes measures were adjusted for age, sex, race, BMI, and activity level. Outcome measures were standardized by subtracting the variable value from the mean and divided by the standard deviation so that all the bone outcomes measured could be compared on a similar scale. Linear trends were examined using generalized estimating equations to account for repeated measures. These analyses were to determine if there were associations between HCV or HCV/HIV status with bone measures, inflammatory biomarkers, and BTMs. Models were adjusted for age at baseline (≤54 vs > 54 years), race (white, black, or other), sex, BMI (normal, overweight, or obese), alcohol use (AUDIT-C negative vs positive score), physical activity (iPAQ- low, moderate or high), and smoking (current smoker Y/N) using all participants with data available (i.e. complete case analysis). In the models which compared bone outcomes in individuals with HCV monoinfection and HIV/HCV co-infection, we conducted sensitivity analyses excluding those participants who switched off from TDF during the study interval or in the year prior to the baseline visit, since switching off from TDF could confound the results. All statistical analyses were performed in SAS v9.4 (Cary, NC).

## Results

Of the 198 participants (127 with HCV) who signed informed consent and had a baseline visit with DXA (**Supplementary Figure S1**), 115 had a follow-up DXA scan (62 with HCV); three of those in the HCV group who received DAA were found to have a detectable HCV at a follow up study visit and were excluded from subsequent analyses. Participant characteristics by HCV status are shown in **Table 1**. Among 59 participants with HCV, median age at screening was 54 years (IQR 49, 59), 23 were female (39%), 10 (17%) were white and 45 were African American (76%), which were similar to the reference population without HIV or HCV. Median BMI was 28.3 kg/m^2^ (25.2, 31.7) and 40 (68%) participants were current smokers. There was a trend to more physical activity in the reference group compared to the HCV group (p=0.06). Median kPa by transient elastography was higher in those with HCV compared to the reference group (6.1 vs 5.5 kPa, p=0.007), with 13% of those with HCV having kPa > 12.5, a value predictive of cirrhosis (40). None of the participants had clinical cirrhosis or known hepatocellular carcinoma. Median baseline HCV viral load was 6.1 log10 IU/L (5.5 log10, 6.6 log10) and 18 (30.5%) participants had HIV-HCV co-infection. Participant characteristics by HCV/HIV status were similar (**Table 2**). Those with HCV-HIV co-infection were more likely to be women, to be less physically active and to have smaller FibroScan Scores. Among the participants with HIV infection, 94% had HIV suppression (viral load <50 cp/ml) with a median CD4 T cell count of 649 (IQR 381, 939) cells/ml. Two participants reported receiving TDF at baseline, one of whom had an HIV RNA of 41,500 cp/mL suggesting non-adherence to the antiretroviral treatment. Three participants switched from TDF to TAF in the 12 months prior to the baseline visit.

**Table 1:** Participant characteristics at baseline by HCV status.

		HCV status			
Characteristic	All (n=112)	Chronic HCV treated (n=59)	Reference (n=53)	p-value	N
Age at Screening, years (median IQR)	54.0 (48.0,58.0)	54.0 (49.0,59.0)	53.0 (48.0,57.0)	0.48	112
Female (n,%)	46 (41.1)	23 (39.0)	23 (43.4)	0.64	112
Race (n,%)				0.21	112
- White	20 (17.9)	10 (16.9)	10 (18.9)		.
- AA	88 (78.6)	45 (76.3)	43 (81.1)		.
- Other	4 (3.6)	4 (6.8)	0 (0.0)		.
BMI (n,%)				0.15	112
- <25	33 (29.5)	13 (22.0)	20 (37.7)		.
- 25-<30	37 (33.0)	23 (39.0)	14 (26.4)		.
- 30+	42 (37.5)	23 (39.0)	19 (35.8)		.
Log10 HCV Viral Load, IU/L (median IQR)	6.1 (5.5, 6.6)	6.1 (5.5, 6.6)	——		56
HIV Positive (n,%)	18 (16.1)	18 (30.5)	0		112
- CD4 count (median IQR)	649.5 (381.0,939.5)	649.5 (381.0,939.5)	——		16
- HIV VL Undetectable (n,%)	15 (93.8)	15 (93.8)	——		16
- ARV includes Tenofovir disoproxil (n,%)	3 (18.8)	3 (18.8)	——		16
- ARV includes Protease Inhibitor (n,%)	0	0	——		18
Smoking Status (n,%)				0.29	112
- Never Smoked	20 (17.9)	9 (15.3)	11 (20.8)		.
- Former Smoker	14 (12.5)	10 (16.9)	4 (7.5)		.
- Current Smoker	78 (69.6)	40 (67.8)	38 (71.7)		.
Alcohol AUDIT-C Positive (n,%)	21 (19.4)	9 (16.1)	12 (23.1)	0.36	108
FibroScan Score, kPa (median IQR)*	5.7 (4.7,7.7)	6.1 (5.0,10.3)	5.5 (4.6,6.1)	0.007	96
Activity Categories (n,%)				0.06	112
- Low	31 (27.7)	22 (37.3)	9 (17.0)		.
- Moderate	44 (39.3)	20 (33.9)	24 (45.3)		.
- High	37 (33.0)	17 (28.8)	20 (37.7)		.
Log10 Total MET Minutes, (median IQR)	3.1 (2.7,3.6)	3.1 (2.7,3.6)	3.3 (2.9,3.6)	0.12	112
Weeks Between Scans (median IQR)	58.1 (54.1,69.1)	60.3 (56.0,71.6)	55.9 (53.1,62.9)	0.009	112
Weeks from DAA end to Final Scan (median IQR) IQRIQR)	46.6 (41.3,53.1)	46.6 (41.3,53.1)			58
Ever Diabetes (n,%)	13 (11.7)	6 (10.2)	7 (13.5)	0.59	111
Vitamin D Supplements Current Use (n,%)	19 (17.0)	11 (18.6)	8 (15.1)	0.62	112
Steroid Current Use > 3 months (n,%)	3 (2.7)	1 (1.7)	2 (3.8)	0.61	111
Dietary Calcium, mg (median IQR)	446.0 (268.8,788.3)	470.0 (247.0,860.0)	434.0 (278.5,742.0)	0.53	112
Hip Fracture of Parent (n,%)	4 (4.9)	4 (10.0)	0 (0.0)	0.05	82

*Missing baseline FibroScan: 5 with HCV, 11 Reference.

**Table 2:** Participant characteristics at baseline by HIV/HCV status.

		HCV HIV Status			
Characteristic	All (n=59)	Monoinfection (n=41)	HIV Coinfection (n=18)	p-value	N
Age at Screening, years (median IQR)	54.0 (49.0,59.0)	56.0 (51.0,61.0)	51.5 (47.0,58.0)	0.07	59
Female (n,%)	23 (39.0)	12 (29.3)	11 (61.1)	0.02	59
Race (n,%)				0.53	59
- White	10 (16.9)	7 (17.1)	3 (16.7)		.
- AA	45 (76.3)	30 (73.2)	15 (83.3)		.
- Other	4 (6.8)	4 (9.8)	0 (0.0)		.
Log10 HCV Viral Load, IU/L (median IQR)	6.1 (5.5,6.6)	6.0 (5.5,6.6)	6.4 (5.7,6.6)	0.60	55
BMI (n,%)				0.74	59
- <25	13 (22.0)	8 (19.5)	5 (27.8)		.
- 25-<30	23 (39.0)	16 (39.0)	7 (38.9)		.
- 30+	23 (39.0)	17 (41.5)	6 (33.3)		.
HIV Positive	18 (30.5)	0	18 (100.0)		59
- CD4 count (median IQR)	649.5 (381.0,939.5)	——	649.5 (381.0,939.5)		16
- HIV VL Undetectable (< 200 copies/mL, n,%)	15 (93.8)	——	15 (93.8)		16
- ARV includes Tenofovir disoproxil (n,%)	3 (18.8)	——	3 (18.8)		16
- ARV includes Protease Inhibitor (n,%)	0	——	0		18
Smoking Status (n,%)				0.14	59
- Never Smoked	9 (15.3)	5 (12.2)	4 (22.2)		.
- Former Smoker	10 (16.9)	5 (12.2)	5 (27.8)		.
- Current Smoker	40 (67.8)	31 (75.6)	9 (50.0)		.
Alcohol AUDIT-C Positive (n,%)	9 (16.1)	8 (20.5)	1 (5.9)	0.25	56
FibroScan Score, kPa (median IQR)	6.1 (5.0,10.3)	7.3 (5.5,10.9)	5.5 (5.0,6.1)	0.05	54
Activity Categories (n,%)				0.03	59
- Low	22 (37.3)	11 (26.8)	11 (61.1)		.
- Moderate	20 (33.9)	15 (36.6)	5 (27.8)		.
- High	17 (28.8)	15 (36.6)	2 (11.1)		.
Log10 Total MET Minutes (median IQR)	3.1 (2.7,3.6)	3.2 (2.8,3.7)	2.7 (2.3,3.3)	0.01	59
Weeks Between Scans (median IQR)	60.3 (56.0,71.6)	59.9 (55.1,65.9)	61.8 (57.1,88.1)	0.07	59
Weeks from DAA end to Final Scan (median IQR) IQR)	46.6 (41.3,53.1)	45.9 (41.0,47.9)	48.0 (43.9,76.6)	0.08	58
Ever Diabetes (n,%)	6 (10.2)	4 (9.8)	2 (11.1)	1	59
Vitamin D Supplements Current Use (n,%)	11 (18.6)	7 (17.1)	4 (22.2)	0.72	59
Steroid Current Use > 3 months (n,%)	1 (1.7)	1 (2.5)	0 (0.0)	1	58
Dietary Calcium, mg (median IQR)	470.0 (247.0,860.0)	403.8 (175.0,942.0)	584.5 (312.0,769.0)	0.97	59
Hip Fracture of Parent (n,%)	4 (10.0)	3 (10.7)	1 (8.3)	1	40

For those with HCV, 15 participants (25%) received ledipasvir/sofosbuvir; 15 (25%), elbasvir/grazoprevir; 12 (20%) glecaprevir/pibrentasvir; 10 (17%) osofosbuvir/velpatasvir; 4 participants (7%) ombitasvir/paritaprevir/ritonavir; and 1 sofosbuvir/velpatsvir/voxilarevir. In two participants, the DAA regimen was not known. One participant who received elbasvir/grazoprevir also received ribavirin. For all participants (both those with HCV and those in the reference group), the median duration between the baseline and follow-up DXA was 58.1 (IQR 54.1, 69.1) weeks; 21.4% (24) had follow-up duration of ≥ 72 weeks. For those with HCV infection, the median time between DXA evaluations was 60.3 weeks (56.0, 71.6) and the median time between end of DAA treatment and the follow-up DXA evaluation was 46.1 (41.1, 52.0) weeks. The one participant with HIV/HCV co-infection receiving suppressive ART with TDF remained on TDF during the study interval. The participant who reported receiving TDF at baseline, despite a high HIV RNA started TAF during the study interval.

### Inflammatory markers at baseline and after HCV Cure: HCV vs reference group

**Figure 1:**
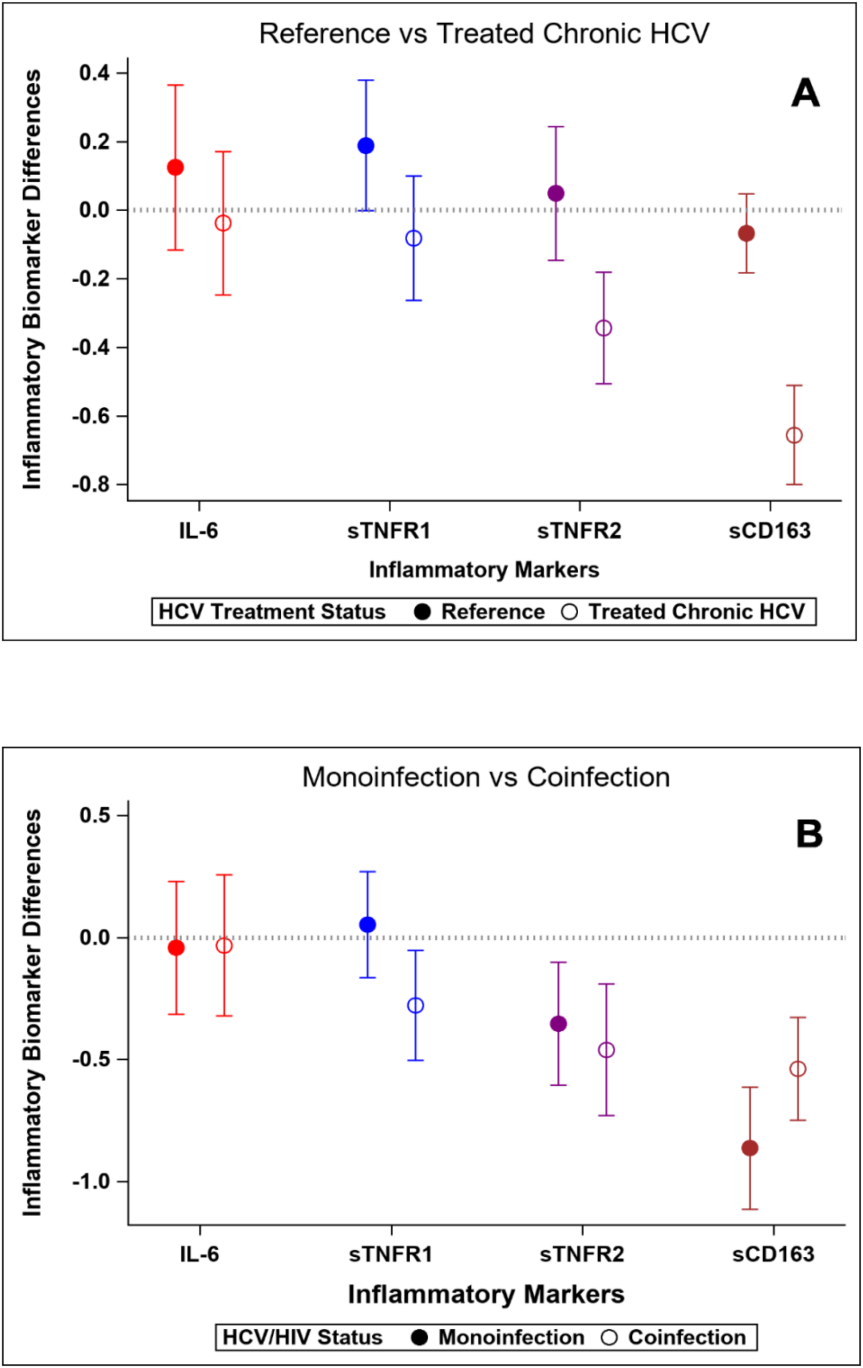
Changes in standardized inflammatory markers over the study interval. Y-axis values represent standardized mean change over time. Positive values indicate greater increase (or smaller decrease) compared to reference group. **(A)** Reference population vs HCV-infected group. **(B)** HCV mono-infected group vs HIV-HCV co-infected group.

At baseline, concentrations of soluble TNFR1, TNFR2 and CD163 were significantly higher in HCV participants compared to the reference group, while IL-6 concentrations were similar between groups (**Table 3**). After treatment with DAA, concentrations of soluble TNFR1, TNFR2 and sCD163 decreased significantly during follow-up in those with HCV (all p<0.0001). The mean percentage change from baseline (95% confidence interval) in the HCV group vs the reference group were: sTNFR1 -3.6% (-10.8, 3.4) vs 6.9% (-1.5, 15.4); sTNFR2 -14.5% (-22.1, -6.7) vs 1.6% (-8.0,12.0); sCD163 -33.2% (-40.0, -26.6) vs -3.9% (-10.4, 3.7). There were no changes in the IL-6 in either the HCV group or the reference group (**Figure 1A**). No difference was observed when comparing the post-DAA inflammatory marker concentrations in the HCV group to concentrations in the reference group (p=0.88-0.99) (**Supplementary Table S1**). In a supplemental analysis (**Supplementary Figure S2**), we compared the changes in inflammatory markers in those with HCV mono-infection to the reference population. We found that concentrations of soluble TNFR2 and sCD163 decreased significantly during follow-up in those with HCV mono-infection; there were no changes in the concentrations of sTNFR1 or IL-6 in either the HCV mono-infected group or the reference group.

**Table 3:** Adjusted baseline inflammatory markers by HCV status.

Characteristic	Chronic HCV treated (n=59)	Reference (n=53)	p-value
IL-6 (pg/mL)	5.74 (3.45, 8.02)	4.68 (1.96, 7.41)	0.33
sTNFR1 (pg/mL)	2005.8 (1711.2, 2300.4)	1724.1 (1372.6, 2075.6)	0.04
sTNFR2 (pg/mL)	5593.7 (4817.8, 6369.6)	4446.7 (3521.0, 5372.4)	0.002
sCD163 (ng/mL)	1248.2 (1010.9, 1485.5)	588.16 (304.99, 871.32)	<0.0001

Inflammatory Markers are adjusted by Age, Sex, Race, BMI, and Activity Level.

### Inflammatory markers at baseline and after HCV Cure: HCV mono-infection vs HIV/HCV co-infection

At baseline, sTNFR1 and sTNFR2 concentrations were higher in participants with HIV/HCV co-infection than those with HCV mono-infection, whereas IL-6 and sCD163 concentrations were similar in the two groups (**Table 4**). After HCV cure, sTNFR1 significantly decreased in those with HIV/HCV co-infection compared to those with HCV mono-infection (p-value=0.04 for difference in slopes between the two groups) (**Figure 1B**). We also observed a trend to a greater decrease in sCD163 concentrations in the HCV monoinfected group, compared to those with HIV/HCV co-infection (p=0.051) (**Figure 1B**).

**Table 4:** Adjusted baseline inflammatory markers by HCV/HIV status.

Characteristic	Mono-infected (n=41)	Co-infected (n=18)	p-value
IL-6 (pg/mL)	5.21 (2.52, 7.90)	5.61 (2.16, 9.06)	0.82
sTNFR1 (pg/mL)	1801.2 (1417.0, 2185.5)	2468.1 (1975.7, 2960.5)	0.008
sTNFR2 (pg/mL)	5362.0 (4496.8, 6227.1)	6499.9 (5391.3, 7608.6)	0.04
sCD163 (ng/mL)	1221.8 (902.39, 1541.2)	1333.1 (923.81, 1742.4)	0.59

Inflammatory Markers are adjusted by Age, Sex, Race, BMI, and Activity Level.

### Bone outcomes at baseline and after HCV cure vs the reference group

**Figure 2:**
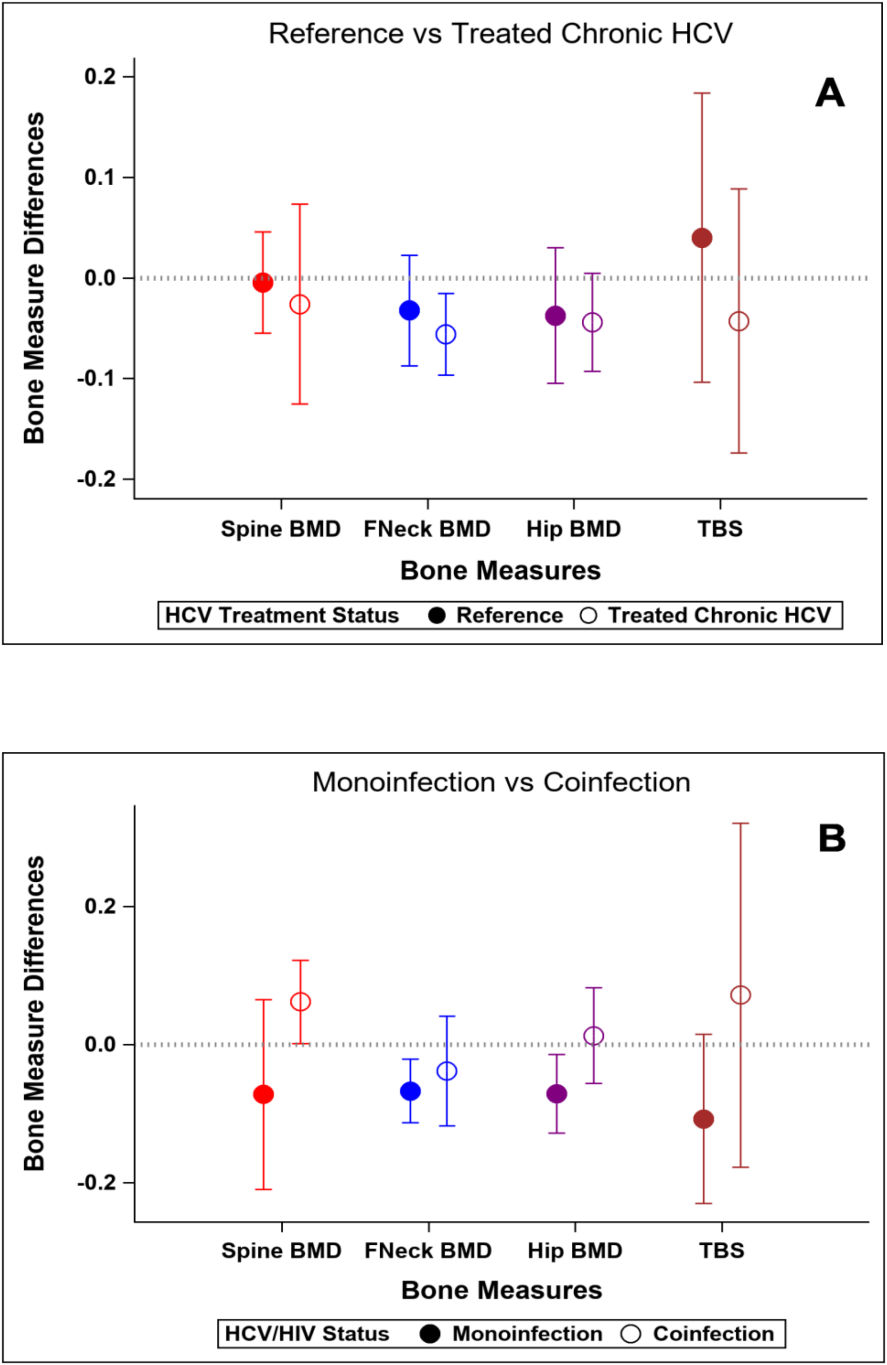
Changes in standardized bone measures over the study interval. Y-axis values represent standardized mean change over time. Positive values indicate greater increase (or smaller decrease) compared to reference group. **(A)** Reference population vs HCV-infected group. **(B)** HCV-monoinfected group vs HIV-HCV co-infected group.

**Figure 3:**
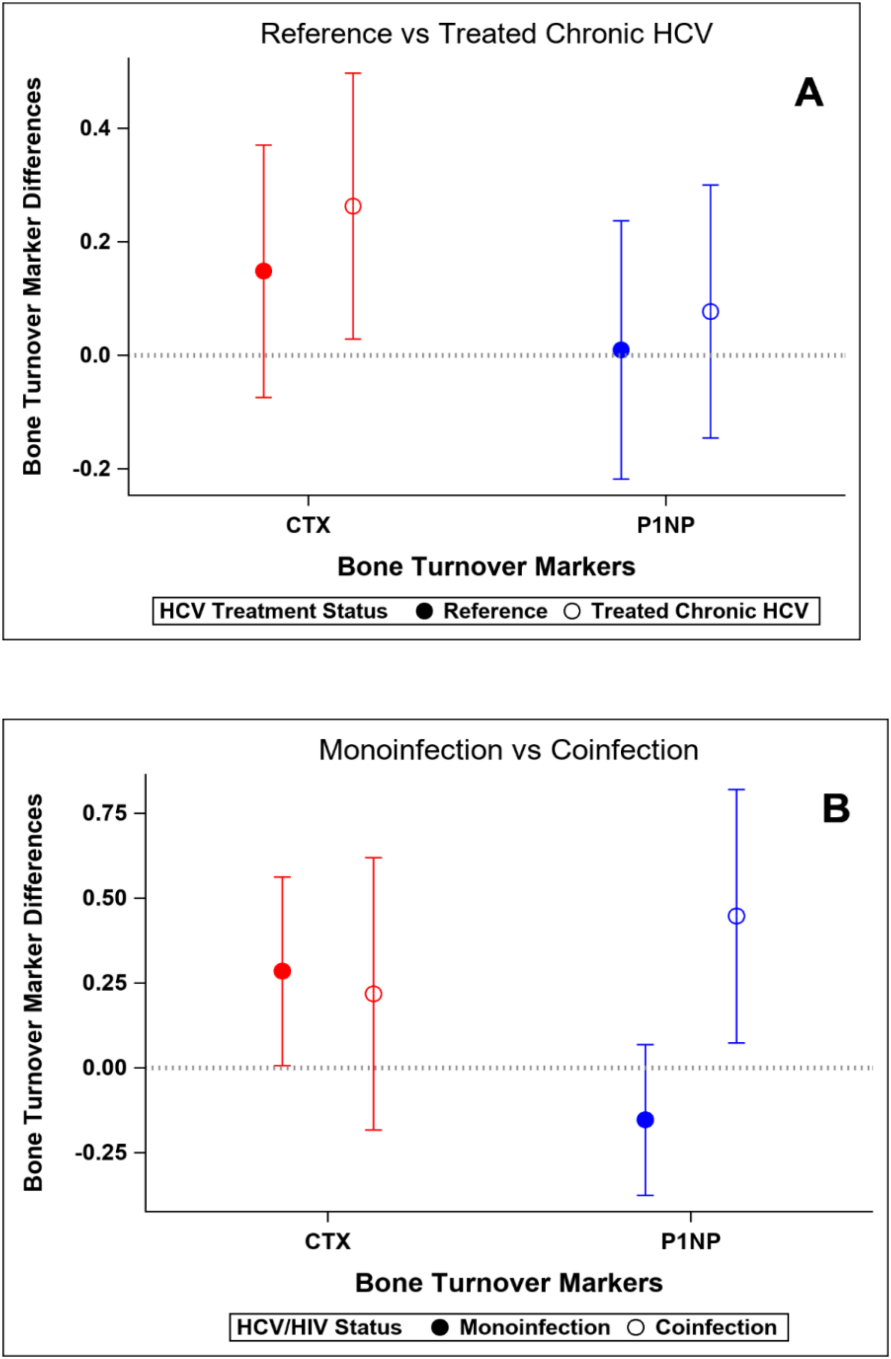
Changes in standardized bone turnover markers over the study interval. Y-axis values represent standardized mean change over time. Positive values indicate greater increase (or smaller decrease) compared to reference group. **(A)** Reference population vs HCV-infected group. **(B)** HCV mono-infected group vs HIV-HCV co-infected group.

At baseline, participants with HCV and those in the reference population did not demonstrate significant differences in bone density measures at the spine or hip, or in bone quality as measured by TBS or BTMs after adjustment for age, gender, race, BMI, and physical activity level (**Table 5**). The prevalence of low BMD at any site (T-score ≤ -1 at either lumbar spine, femoral neck, or total) was similar in those with and without HCV (34% vs 28%; p=0.52); the prevalence of osteoporosis (T-score ≦ -2.5) was low (5% vs 2%, respectively; p=0.62). After HCV cure, no significant differences were observed in changes in BMD, TBS (**Figure 2A**), or BTMs (**Figure 3A**) between those with HCV and the reference population. The upper bound of the 95% confidence interval for the difference in standardized BMD between the groups likely excludes a clinically relevant effect of HCV cure during the follow-up period (spine: 0.089, femoral neck: 0.04, total hip: 0.076) (41). Results were similar when the HCV group was restricted to those with HCV mono-infection (**Supplementary Figures S3**, **S4**).

**Table 5:** Adjusted bone measures by HCV status at baseline.

Characteristic	Chronic HCV treated (n=59)	Reference (n=53)	p-value
Lumbar Spine BMD (g/cm^2^), mean (95% CI)^*^	1.08 (1.01, 1.14)	1.06 (0.98, 1.14)	0.68
Lumbar Spine T-score, median (Q1, Q3)	0.12 (-0.74, 1.61)	0.05 (-0.92, 0.93)	0.41
Femoral Neck BMD (g/cm^2^), mean (95% CI)^*^	0.82 (0.76, 0.88)	0.83 (0.76, 0.90)	0.64
Femoral Neck T-score, median (Q1, Q3)	0.25 (-1.06, 1.23)	0.00 (-0.50, 1.12)	0.93
Total Hip BMD (g/cm^2^), mean (95% CI)^*^	0.99 (0.94, 1.04)	1.00 (0.94, 1.06)	0.74
Total Hip T-score, median (Q1, Q3)	0.59 (-0.48, 1.34)	0.46 (0.04, 1.16)	0.88
Low BMD at any site (T-score ≤ -1), n (%)	20 (34%)	15 (28%)	0.52
Osteoporosis at any site (T-score ≤ -2.5), n (%)	3 (5%)	1 (2%)	0.62
Trabecular Bone Score (g/cm^2^), mean (95% CI)^*^	1.35 (1.31, 1.39)	1.33 (1.28, 1.38)	0.41
CTX, (pg/ml), median (Q1, Q3)	410.00 (280.00, 545.00)	455.00 (340.00, 630.00)	0.12
P1NP, (ug/ml), median (Q1, Q3)	59.37 (43.80, 84.84)	63.52 (45.68, 87.20)	0.86

Bone Measures are adjusted by Age, Sex, Race, BMI, and Activity Level.

### Bone outcomes at baseline and after HCV cure: HCV mono-infection vs HIV/HCV co-infection groups

At baseline, median T-scores were lower in those with HIV/HCV co-infection compared to HCV mono-infection at the lumbar spine (0.66 vs -0.39; p=0.009) and total hip (0.79 vs -0.07; p=0.04), but similar at the femoral neck. The bone formation marker, P1NP, tended to be lower in those with HIV/HCV co-infection (p=0.09) (**Table 6**). After HCV cure, lumbar spine BMD tended to increase in those with HIV/HCV co-infection group, compared to the mono-infection group (difference between groups (0.13; 95% confidence interval (CI) -0.02, 0.28; p=0.08). At the total hip, BMD also tended to increase in those with HIV/HCV co-infection compared to the mono-infected population, with a difference in T-score between groups (0.08; 95% confidence interval (CI) -0.01, 0.17; p=0.07). However, no difference was observed between the groups in the changes in femoral neck BMD (p=0.54). (**Figure 2B**). No differences were observed in the changes in TBS between the groups. The changes following HCV cure in the bone resorption marker, CTX, were similar among the two groups with HCV infection, whereas the bone formation marker, P1NP, significantly increased in the group with HIV/HCV co-infection compared to the group with HCV mono-infection (p=0.007 for differences in the changes in P1NP by group) (**Figure 3B**). The mean percentage changes from baseline (95% confidence interval) in the HCV mono-infected group vs the HIV/HCV co-infected group were: CTX -16.0% (-1.4, 36) vs -13.8% (-7.3, 43.4); P1NP -7.4% (-18.1, 6.7) vs 26.5% (3.2, 59.2).

**Table 6:** Adjusted measures by HCV/HIV status at baseline.

Characteristic	Monoinfected (n=41)	Coinfected (n=18)	p-value
Lumbar Spine BMD (g/cm^2^), mean (95% CI)^*^	1.10 (1.02, 1.18)	1.01 (0.90, 1.11)	0.10
Lumbar Spine T-score, median (Q1, Q3)	0.66 (-0.31, 2.15)	-0.39 (-1.42, 0.12)	0.009
Femoral Neck BMD (g/cm^2^), mean (95% CI)^*^	0.81 (0.74, 0.87)	0.80 (0.72, 0.89)	0.96
Femoral Neck T-score, median (Q1, Q3)	0.44 (-0.80, 1.21)	-0.33 (-1.02, 1.56)	0.39
Total Hip BMD (g/cm^2^), mean (95% CI)^*^	1.01 (0.95, 1.07)	0.96 (0.88, 1.04)	0.20
Total Hip T-score, median (Q1, Q3)	0.79 (0.01, 1.34)	-0.07 (-0.69, 0.98)	0.04
Low BMD at any site (T-score ≤ -1), n (%)	12 (29%)	9 (45%)	0.20
Osteoporosis at any site (T-score ≤ -2.5), n (%)	3 (7%)	0	0.54
Trabecular Bone Score (g/cm^2^), mean (95% CI)^*^	1.34 (1.29, 1.39)	1.32 (1.25, 1.38)	0.47
CTX, (pg/ml), median (Q1, Q3)	410.00 (280.00, 550.00)	405.00 (320.00, 560.00)	0.92
P1NP, (ug/ml), median (Q1, Q3)	66.27 (45.45, 97.13)	54.44 (43.00, 71.23)	0.09

Bone Measures are adjusted by Age, Sex, Race, BMI, and Activity Level.

Given the apparent differences in the changes in lumbar spine BMD, total hip BMD, and P1NP concentrations, we repeated the analysis excluding the 3 participants with HIV infection who switched off from tenofovir disoproxil fumarate in the year prior to the baseline DXA and similar results were observed (point estimates for between group T-score difference: lumbar spine, 0.14; total hip, 0.08; femoral neck: 0.02; mean percentage change in P1NP in the HIV/HCV co-infected group: 32.8%). We repeated this analysis, excluding in addition the individual who reported TDF use at baseline but had a high RNA (suggesting non-adherence to the antiretroviral treatment) and then started TAF during follow-up. Excluding these 4 participants yielded similar results to the primary analysis (point estimates for between group T-score difference: lumbar spine, 0.13; total hip, 0.08; femoral neck; 0.03; mean percentage change in P1NP in the HIV/HCV co-infected group: 30.8%) regarding all bone outcomes after HCV cure.

## Discussion

To our knowledge, this is the first longitudinal study to evaluate the effect of HCV cure with DAA on systemic inflammation and bone outcomes (density, quality, and turnover) in participants with HCV infection, with and without concomitant HIV infection. Since HCV chronic infection has been associated with lower BMD and a 1.4-3.6-fold increased fracture rate in previous studies (14, 18–20) which is thought to be mediated by increased systemic inflammation (19), we hypothesized that HCV eradication would improve metabolic bone health by reversing the impact of persistent viremia and inflammation. We found that, despite clear decreases in markers of systemic inflammation, HCV cure was not associated with significant changes in bone density, quality (as measured by TBS), or turnover over period of 1–2 years. Our findings suggest that, unlike other extra-hepatic manifestations of HCV that may improve or present a reduced risk, metabolic bone disease is not significantly impacted in the 1–2 years following HCV cure.

Increased systemic inflammation is thought to be a driver of bone loss in both HCV and HIV infection. In *in vitro* studies, tumor necrosis factor (TNF)-α has been shown to inhibit osteoblast differentiation (42) and promote osteoclast survival (43), which may lead to net bone resorption and bone loss. In our study, individuals with chronic HCV prior to DAA had higher pre-treatment concentrations of soluble TNF- α receptors compared to the reference population, which were particularly elevated in those with HIV/HCV co-infection. Similar to other studies (44–50) examining the effects of HCV cure on systemic inflammation, results were variable depending on the marker studied; in our study, we found decreases in soluble TNF-α receptor concentrations after successful DAA treatment, particularly sTNFR2. In addition, we observed significant decreases in the monocyte activation marker, sCD163, with a greater decrease in those with HCV mono-infection, indicative of residual inflammation related to concomitant HIV infection, similar to the CHAMPS study (44).

Despite these decreases in systemic inflammation with HCV cure, we observed no significant improvement in bone outcomes in those with HCV compared to the reference population over the 1–2 years of observation after DAA initiation. Consistent with the effects of aging on bone, BMD tended to decrease and bone resorption tended to increase over the study interval. The reasons behind this apparent paradox in which we observed changes in systemic inflammation without changes in bone outcomes are unclear, but it is possible that circulating markers of inflammation may not be representative of local inflammation within the bone microenvironment. These results may also suggest that in the context of HCV cure the bone effects of chronic HCV may not be reversible over this 2-year period, something that could be related to structural damage to bone architecture, osteoblast or osteocyte senescence, or lasting epigenetic modifications induced by chronic inflammation. Alternatively, these findings could indicate that there is no direct or indirect effect of HCV infection on BMD.

Multiple studies have demonstrated low BMD and abnormal bone microarchitecture in persons with HCV (15, 22, 51). It is possible that differences in bone parameters between populations with and without HCV, which have been attributed to HCV infection, may be related to unmeasured confounders affecting bone health such as nutrition, lifestyle, physical activity or body composition. Indeed, our reference group was drawn from the same underlying population as those with HCV and were very similar in all respects, including on bone health parameters. Taken together, our results confirm that increased systemic inflammation may not be immediately related to bone health in HCV (15) and, importantly, our findings suggest that HCV cure may not improve bone health in those with HCV.

In contrast with our overall findings, we found that individuals with HIV/HCV co-infection tended to have increases in lumbar spine BMD and total hip BMD after HCV cure, compared to individuals with HCV mono-infection, accompanied by significant increases in the osteoblast marker, P1NP. In populations with HIV, HCV co-infection is a risk factor for both osteoporosis and fragility fracture. In a meta-analysis of 15 studies, Dong et al. found that persons with HCV/HIV co-infection were 63% more likely to have osteoporosis and 77% more likely to have fractures, compared to those with HIV mono-infection (21). Previous studies examining the impact of HCV cure on bone parameters in persons with HIV/HCV co-infection have been mixed and have focused on earlier regimens, containing interferon-α and/or ribavirin. In an ACTG study, SVR with interferon-α and ribavirin was associated with significant decreases in BTMs (27). In a Spanish study, persons with HIV/HCV co-infection who achieved SVR with treatment regimens that included interferon and/or ribavirin showed no differences in the change in BMD compared to the 39% of the population, who did not achieve SVR (30).

Our study extends these findings on the bone effects of HCV treatment in persons with HIV co-infection by focusing on persons who achieved SVR with interferon and ribavirin-free regimens (except for one participant who received ribavirin with DAA), in that interferon and ribavirin may have direct effects on bone metabolism (32, 33). We also were able to directly compare the impact of HCV cure with DAA in people with HCV mono-infection and HIV/HCV co-infection. It should be noted however that our sample size of persons with HIV/HCV co-infection was small, since many people with HIV/HCV co-infection had already been treated with DAA in our clinic when our study began, and those that did enroll were among the most difficult to engage in care. Also of note, while the magnitude of the difference in the change in standardized BMD (equivalent to a T-score or Z-score) between those with HCV mono-infection and HIV/HCV co-infection appears to be small (0.08-0.13), it was similar to the effect of bisphosphonates on T-score over 1 year from a recent clinical trial in post-menopausal women (41). We were concerned that the observed effect on BMD and bone turnover was being driven by persons who switched off from tenofovir disoproxil fumarate immediately prior to baseline or during follow up, but excluding these participants did not appreciably change the results. It should be emphasized that the sample size of people with HIV/HCV coinfection who switched off from TDF was small, limiting a more in-depth assessment of this potentially important confounder. Larger longitudinal studies should examine the effect of DAA on bone parameters in persons with HIV/HCV co-infection and examine the potentially confounding effect of TDF switching, especially in resource limited settings where TDF continues to be widely used.

Our study has some additional limitations. First, the study duration was relatively short, but generally treatment effects on bone are seen within the first 6 months, such as switching off bone-toxic antiretroviral therapies or bone-specific pharmacologic interventions. Longer studies may be warranted. Second, the population of persons with HCV was generally difficult to treat as evidenced by a large number of people who were lost to follow up and/or never started DAA. It is possible that a study in a different population may have yielded different results. Next, our reference population without HCV or HIV was recruited by flyers in local clinics and by word of mouth, mostly referred by other participants. While we believe that this group was drawn from the same underlying population as those with HCV, there may have been differences that introduced unmeasured bias. Finally, our sample size was small, especially in those with HIV/HCV co-infection; larger studies are needed.

In conclusion, our study indicates that HCV cure does not seem to have an impact on bone parameters in those with HCV mono-infection, despite decreases in systemic inflammation. Further study is needed in individuals with HIV/HCV co-infection to understand if the trends towards improved spine and hip BMD that we observed are confirmed. Nevertheless, given the consistent finding of lower BMD in persons with HIV/HCV co-infection compared to HIV mono-infection, our data support improved efforts to promote DXA screening in persons with HIV and concomitant HCV infections in order to identify and treat metabolic bone disease and therefore prevent future fractures.

## Data Availability

The raw data supporting the conclusions of this article will be made available by the authors, without undue reservation.

## Supplementary material

Supplementary Figure 1 Study flow.

Supplementary Figure 2 Changes in Standardized Inflammatory Markers over the Study Interval: Reference population vs HCV mono-infected group.

Supplementary Figure 3 Changes in Standardized Bone Measures over the Study Interval. Reference population vs HCV mono-infected group.

Supplementary Figure 4 Changes in Standardized Bone Turnover Markers over the Study Interval: Reference population vs HCV mono-infected group.
