# Supplementary material for: Impact of HCV cure on systemic inflammation and bone density, quality, and turnover
Source: Front Immunol. 2025 Nov 28;16:1626875. doi: 10.3389/fimmu.2025.1626875 (PMC12698625; doi:10.3389/fimmu.2025.1626875)
Supplement: Supplementary Figure 1 — Study flow. [file Image1.pdf]

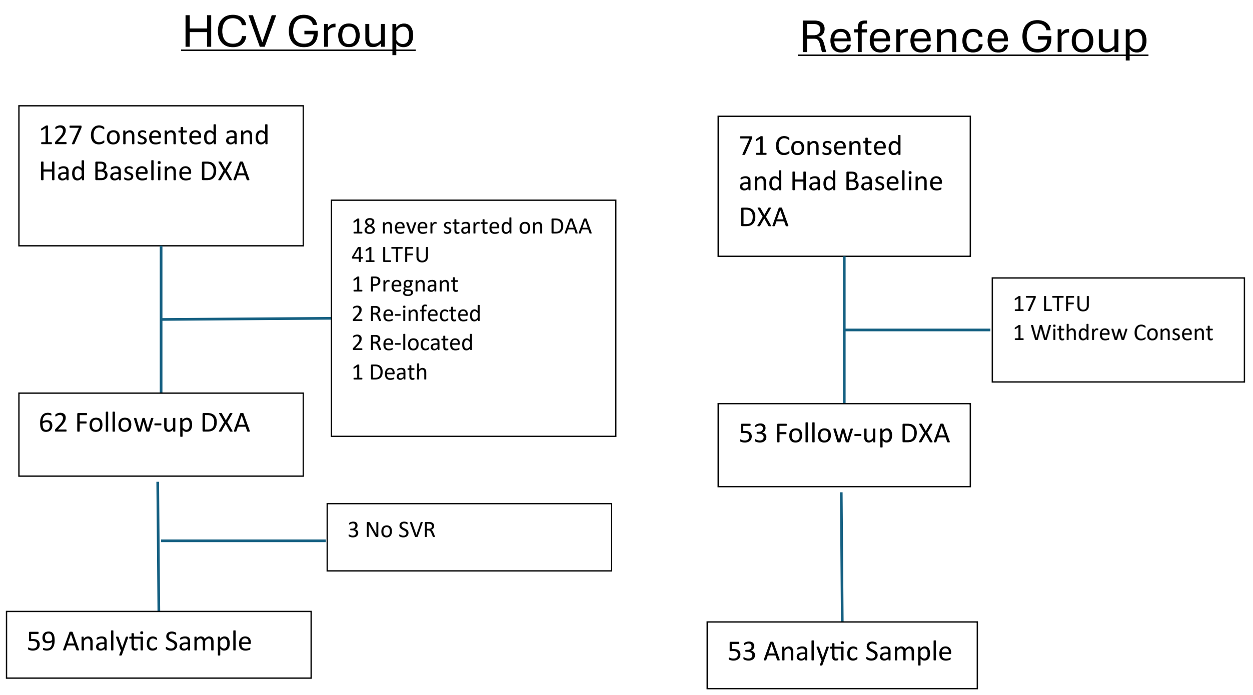

DXA: dual energy X-ray absorptiometry; DAA: direct-acting antiviral; LTFU: lost to follow-up; SVR: sustained virologic response

Supplemental Figure 1. Study Flow
