# Supplementary material for: Impact of HCV cure on systemic inflammation and bone density, quality, and turnover
Source: Front Immunol. 2025 Nov 28;16:1626875. doi: 10.3389/fimmu.2025.1626875 (PMC12698625; doi:10.3389/fimmu.2025.1626875)
Supplement: Supplementary Figure 3 — Changes in Standardized Bone Measures over the Study Interval. Reference population vs HCV mono-infected group. [file Image3.pdf]

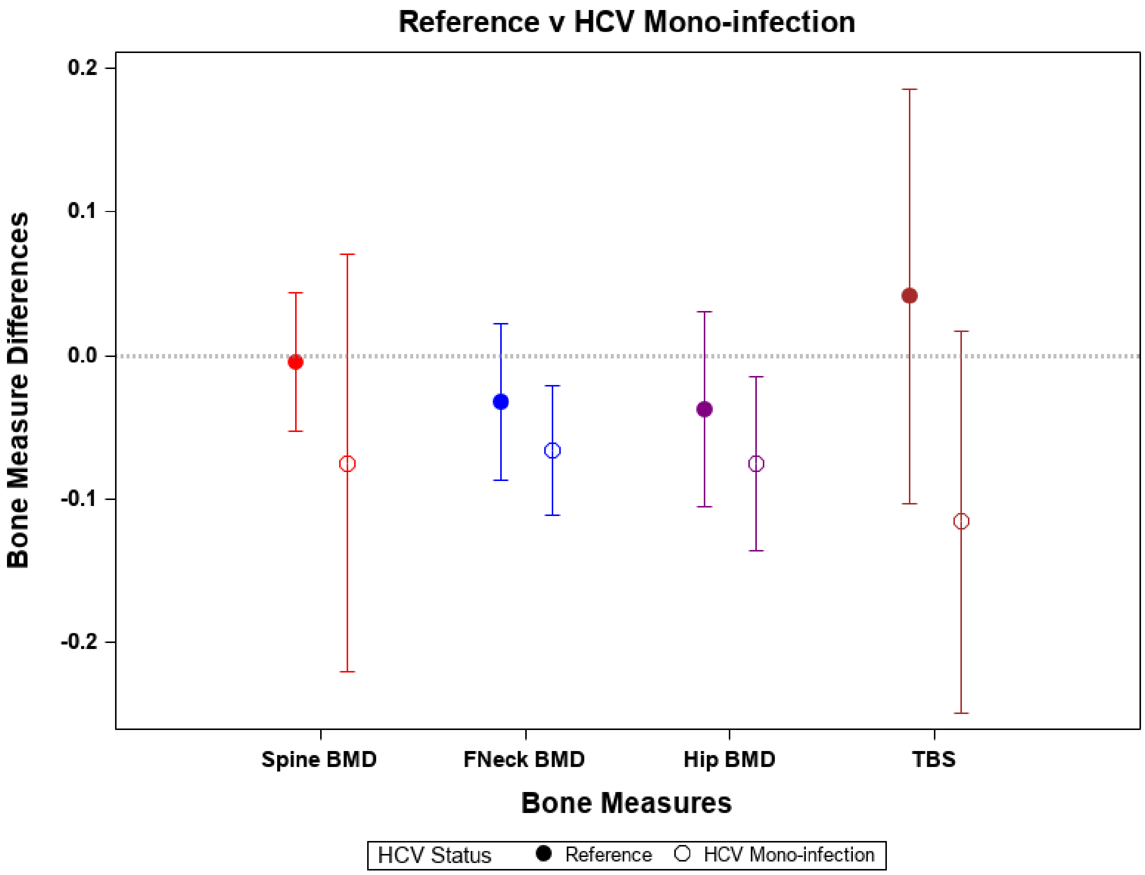

Supplemental Figure 3. Changes in Standardized Bone Measures over the Study Interval. Reference population vs HCV mono-infected group.
