# Supplementary material for: Impact of HCV cure on systemic inflammation and bone density, quality, and turnover
Source: Front Immunol. 2025 Nov 28;16:1626875. doi: 10.3389/fimmu.2025.1626875 (PMC12698625; doi:10.3389/fimmu.2025.1626875)
Supplement: Supplementary file 5 [file Table1.pdf]

Characteristic	Reference Group at Final (n=53)	Chronic HCV Treated at Final (n=59)	p-value
IL6 (pg/ml)	4.68 (1.96, 7.41)	5.57 (2.19, 8.96)	0.95
sTNFR1 (pg/ml)	1724.1 (1372.6, 2075.6)	1679.8 (1276.6, 2082.9)	0.99
sTNFR2 (pg/ml)	4446.7 (3521.0, 5372.4)	4222.8 (3005.6, 5440.1)	0.97
sCD163 (ng/ml)	588.16 (304.99, 871.32)	756.06 (462.99, 1049.1)	0.86

Supplemental Table 1. Comparison of post-direct acting antiviral treatment inflammatory marker concentrations in HCV group vs reference group.
