# Supplementary material for: Impact of HCV cure on systemic inflammation and bone density, quality, and turnover
Source: Front Immunol. 2025 Nov 28;16:1626875. doi: 10.3389/fimmu.2025.1626875 (PMC12698625; doi:10.3389/fimmu.2025.1626875)
Supplement: Supplementary Figure 4 — Changes in Standardized Bone Turnover Markers over the Study Interval: Reference population vs HCV mono-infected group. [file Image4.pdf]

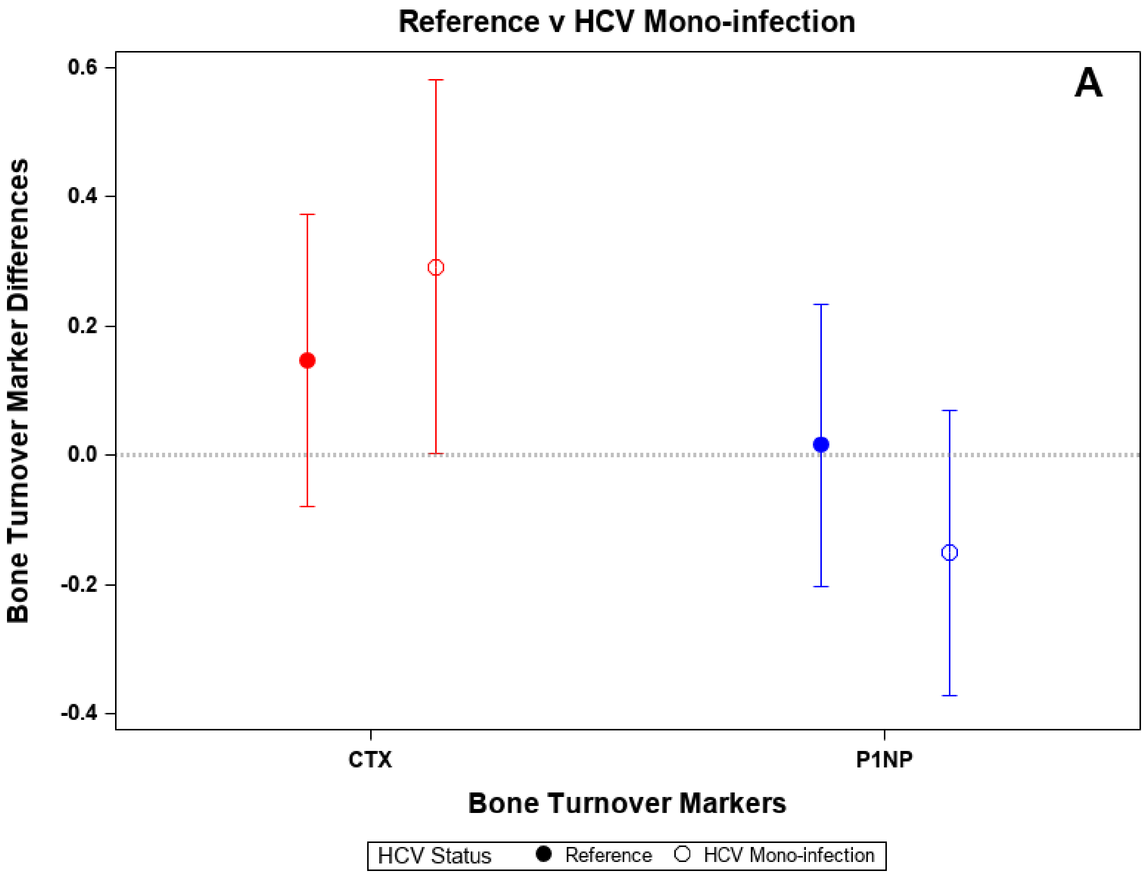

Supplemental Figure 4. Changes in Standardized Bone Turnover Markers over the Study Interval: Reference population vs HCV mono-infected group.
